# Does treatment of short or stunted children aged 6–59 months for severe acute malnutrition using ready to use therapeutic food make them overweight? Data from Malawi

**DOI:** 10.1186/s13690-018-0321-1

**Published:** 2018-12-13

**Authors:** Paul Binns, Mark Myatt

**Affiliations:** Brixton Health, Llwyngwril, Wales

**Keywords:** SAM, CMAM, MUAC, Triceps skinfold thickness, RUTF, Stunting, Overweight

## Abstract

**Background:**

Using mid-upper arm circumference (MUAC) to identify severe acute malnutrition (SAM) tends to identify younger and stunted children compared to alternative anthropometric case-definitions. It has been asserted by some experts, without supporting evidence, that stunted children with low MUAC may have normal weight for height and treatment with ready to use therapeutic food (RUTF) will cause excess adiposity, placing the child at risk for non-communicable diseases (NCD) later in life. It is recommended that children aged less than 6 months should not be treated with RUTF. Height cut-offs are frequently used in SAM treatment programmes to identify children likely to be aged less than 6 months and thus not eligible for treatment with RUTF. This is likely to exclude some stunted children aged 6 months or older. This study examined whether stunted children aged 6 months or older with SAM, identified by MUAC, and treated with RUTF were overweight or had excess adiposity when discharged cured with a MUAC of greater than 125 mm.

**Methods:**

Data was collected at Ministry of Health primary health care facilities delivering community based management of acute malnutrition (CMAM) services between February 2011 and March 2012 in Lilongwe District, Malawi on 258 children aged between 6 and 59 months enrolled in outpatient treatment for SAM with a MUAC less than 115 mm without medical complications irrespective of height on admission. 163 children were discharged as cured when MUAC was 125 mm or greater and there was an absence of oedema and the child was clinically well for 2 consecutive visits. MUAC, triceps skin fold (TSF) thickness and weight were measured at each visit. Height was measured on admission and discharge.

**Results:**

No study subjects (*n = 0*) were overweight or had excess adiposity when discharged cured with a MUAC greater than 125 mm.. There was a tendency towards a higher TSF-for-age (TSF/A) z-scores for severely stunted children compared to non-stunted children (Kruskal-Wallis chi-squared = 9.0675, *p*-value = 0.0107). For children admitted with a height less than 65 cm and those with a height of 65 cm or greater, there was no significant difference in TSF/A z-scores on discharge (Kruskal-Wallis chi-squared = 0.9219, *p* = 0.3370) or AFI/A z-scores on discharge (Kruskal-Wallis chi-squared = 0.0740, *p* = 0.7855).

**Conclusions:**

These results should allay concerns that children aged 6 months and older and with a height less than 65 cm or with severe stunting will become overweight or obese as a result of treatment with RUTF in the outpatient setting using recommended MUAC admission and discharge criteria.

**Trial Registration:**

ISRCTN 92405176 Registered 15th May 2018. Retrospectively registered.

## Background

For determining eligibility for the treatment of severe acute malnutrition (SAM) in selective feeding programmes, a MUAC of less than 115 mm for children aged 6 to 59 months is recommended [1] and increasingly used as a stand alone criterion for admission in national therapeutic feeding guidelines [2]. The exclusion of children from treatment with a mid-upper arm circumference (MUAC) less than 115 mm but with a height of less than 65 cm (or 67 cm) is widespread [3, 4] despite current guidelines from the World Health Organisation (WHO) [5] not including the use of height exclusion criteria. The height cut-offs are used as a proxy for age of 6 months and infants aged less than 6 months with SAM should be hospitalised and are not eligible for treatment with ready to use therapeutic foods (RUTF) [6]. Programmes to treat SAM often operate in settings in which low birth weight, small for gestational age and stunting are common and these cut-offs will often exclude some children considerably older than 6 months [7, 8].

It is well known that using MUAC tends to identify younger children and those who are stunted (i.e. compared to alternative anthropometric case-definitions of SAM such as weight-for-height z-scores (WHZ) less than − 3) [7–9]. A concern is that younger stunted children may not actually be wasted since the shorter limb length and smaller muscle mass could account for the low MUAC (i.e. the child may have a normal weight for height) and that treatment with RUTF may lead to the child becoming overweight [10]. It is an issue of ongoing concern among some practitioners regarding the prognosis of stunted children recruited to selective feeding programmes using MUAC [11] and the potential for placing the child at risk for non-communicable diseases later in life [12, 13].

It has also been postulated that increased consumption of milk, dairy products and other animal proteins during early childhood in non-malnourished children may lead to accelerated growth and obesity and that intakes of these products should be restricted in order to reduce the risk of non-communicable diseases in adulthood [14]. This view is promulgated despite evidence to the contrary indicating that restricting fat intake in children may be counterproductive [15, 16]. It has been argued the use of RUTF, containing sugars, vegetable oil and dried skimmed milk (DSM), at quantities significantly above normal energy requirements for early childhood would appear to be a risk factor for placing children at risk of poor health in later life. In contrast, a further review of the available data concluded, “*observational evidence does not support the hypothesis that dairy fat or high-fat dairy foods contribute to obesity or cardiometabolic risk, and suggests that high-fat dairy consumption within typical dietary patterns is inversely associated with obesity risk*” [17]. A more recent study indicated that children with moderate acute malnutrition treated with lipid based nutrition products mainly gain lean mass [3]. However, concerns about using RUTF in short or stunted children persist and we address this issue with data from a SAM treatment programme.

Metabolic syndrome has been defined in children, adolescents and adults as a cluster of dangerous risk factors for heart disease including hyperglycaemia, hyperlipidaemia and increased blood pressure and is defined by the International Diabetes Federation (IDF) [18]. However, this has only been defined from the age of 6 years upwards due to insufficient data from younger children [19] . Obesity is, however, considered to be a requisite condition to define metabolic syndrome for all age groups in which it has been defined to date.

### Objectives

The objectives of this study were to describe the changes in anthropometry in a cohort of children aged 6–59 months following outpatient treatment for SAM with RUTF and to examine the whether stunting or shortness on admission leads to excess fat deposition using WHO growth standards for triceps skinfold-for-age and estimating the arm fat index developed from reference data from healthy children.

## Methods

Data was collected at Ministry of Health primary health care facilities delivering CMAM services between February 2011 and March 2012 in Lilongwe District, Malawi, on 258 children enrolled in outpatient treatment for SAM aged 6 to 59 months with a MUAC less than 115 mm, without medical complications.

All children aged 6 months or older were eligible for enrolment irrespective of their height. At each visit the child was given a ration of RUTF between 180 and 200 kcal / kg / day. The dosage for all medicines and RUTF were given according to the Malawi National Guidelines for the treatment of SAM [6]. The child was discharged as cured when a MUAC of 125 mm or greater was obtained for 2 consecutive clinic visits, oedema was absent and the child was clinically well. Details of the study have been published elsewhere [20].

Of the 258 children enrolled in treatment 163 (63.2%) were discharged cured and form the study group. From those enrolled with low MUAC and discharged cured (*n* = 163), 23.9% (*n* = 39) were also below – 3 WHZ on admission and 2.4% (*n = 4*) had bilateral pitting (nutritional) oedema on admission .

### Data collection and analysis

Study data analysis was performed for cured cases only. MUAC was measured to the nearest millimetre using a standard non-elastic MUAC tape (UNICEF supply code S0145620 “MUAC, Child 11.5, Red / PAC-50”). On admission and discharge the child’s height was measured to the nearest millimetre using a standard paediatric height board (supine length was measured for children below 24 months of age). Triceps skinfold thickness (TSF) was measured twice at each visit, to the nearest 0.2 mm, using Harpenden® callipers (Holtain Ltd. Crymych, Wales) on a vertical fold of skin at the same position (i.e. the mid-point) on the upper arm as the MUAC measurement. The mean of the two TSF measurements was used in subsequent analyses. Callipers were checked and zeroed at each use and calibrated every 3 months using a Harpenden® 10 mm metal calibration block. The study was completed before the recommended full 2-yearly calibration service was required. MUAC and TSF were measured by the same observer.

Data were collected on paper forms designed for the purpose and were entered into an EpiData Version 3.1 (EpiData Association, Odense, Denmark) database using both interactive checking for range and legal values and double-entry and validation.

Height-for-age (HAZ), weight-for-age (WAZ), weight-for-height (WHZ) z-scores and triceps skinfold-for-age (TSF/A) z-scores were calculated according to the WHO child growth standards (WGS) [21]. The WHO case definition for overweight and obesity for children aged less than 5 years is a WHZ greater than 2 z-scores above the reference median and greater than 3 z-scores above the reference median respectively [21].

The arm fat index (AFI) was derived by calculation using the method of Rolland-Cachera [22].

$$ {\displaystyle \begin{array}{c}\mathrm{Total}\ \mathrm{Upper}\hbox{-} \mathrm{arm}\ \mathrm{Area}\ \left(\mathrm{TUA}\right)=\frac{{\mathrm{MUAC}}^2}{4\uppi}\\ {}\mathrm{Upper}\hbox{-} \mathrm{arm}\ \mathrm{Fat}\ \mathrm{Area}\ \left(\mathrm{UFA}\right)=\mathrm{MUAC}\times \frac{\mathrm{TSF}}{2}\\ {}\mathrm{Arm}\ \mathrm{Fat}\ \mathrm{Index}\ \left(\mathrm{AFI}\right)=\frac{\mathrm{UFA}}{\mathrm{TUA}}\times 100\end{array}} $$

The measurement of the upper arm fat areas (UFA) using this method has been verified as being closer to measurements obtained through magnetic resonance imaging (MRI) than the method of Jelliffe and Gurney [23, 24] which assumes the arm to be cylindrical leading to an underestimation of fat mass.

Arm fat index for age (AFI/A) z-scores were obtained from age and sex disaggregated data for healthy French children aged between 1 month and 17 years collected in 1994 and reported by Rolland-Cachera [22].

Statistical analysis was performed using the *R* Language for Data Analysis and Graphics [25]. The Kruskal-Wallis non-parametric test was used for comparisons of TSF/A and AFI/A at discharge by categories of stuntedness at admission and categories of height at admission for cured cases. The *null hypothesis* for the Kruskal-Wallis test is that the data in each group have the same distribution of the variable of interest.

### Limitations

In the absence of an international reference, AFI/A z-scores used in this article were derived from references derived from data from healthy French children collected as part of an international study of growth [26]. The stipulation of healthy “normal” children used to create this reference is not the same as the stipulation of “living in conditions favourable to growth” that was used for the WHO Growth Standards [21]. Both references are, however, criterion-referenced selecting for good health and differences between the AFI/A references used and a hypothetical WGS reference for AFI/A are unlikely to be large [27] .

## Results

**Fig. 1:**
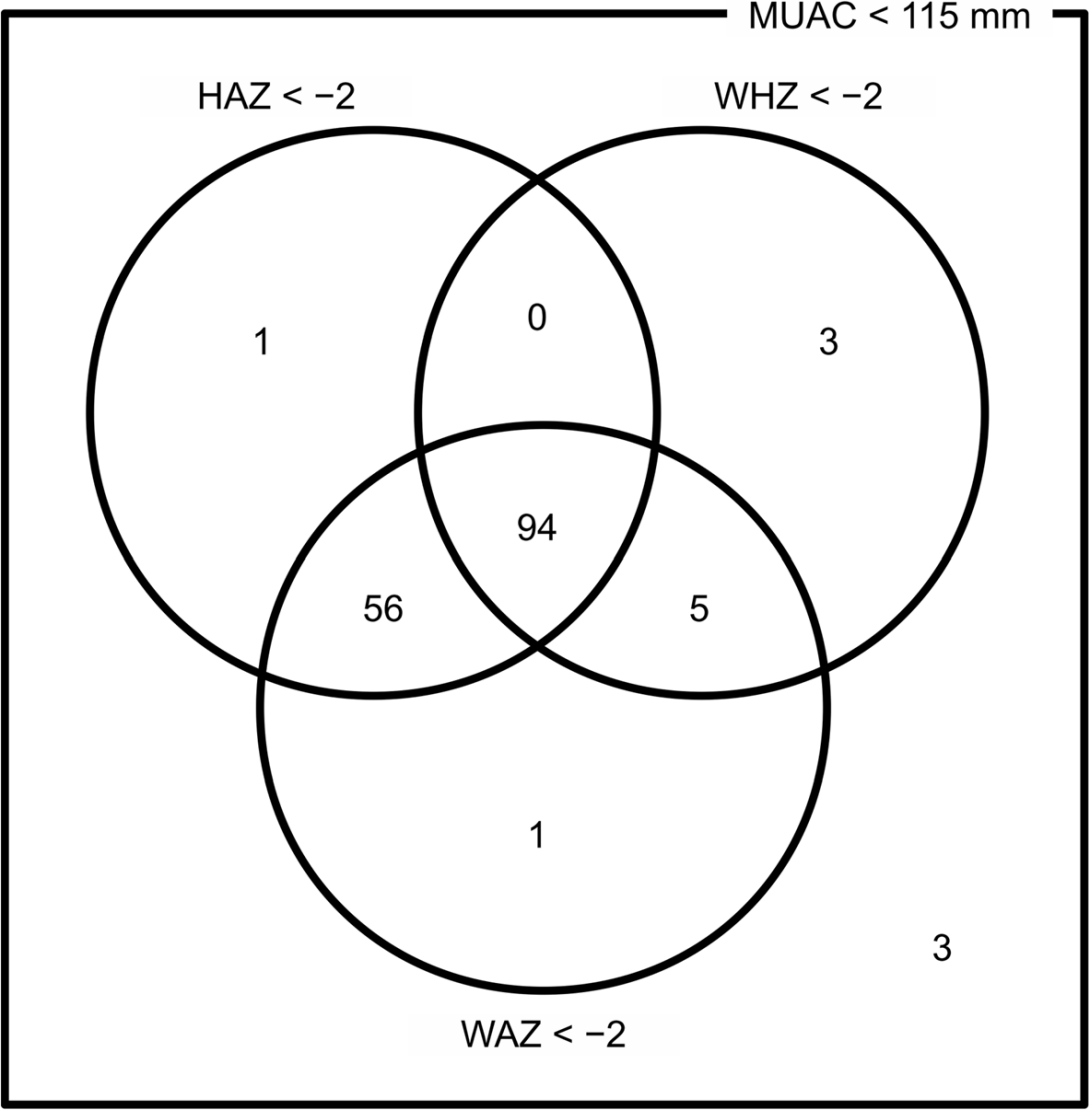
Anthropometric deficits at admission in 163 cured SAM cases (Lilongwe District, Malawi Feb 2011 to March 2012)

Figure 1 summarises the anthropometric data of the 163 study subjects at admission in a Venn diagram [28] in terms of HAZ, WAZ and WHZ according to the World Health Organisation Growth Standards [21]. All study subjects had a MUAC < 115 mm on admission. The majority of the study cohort was undernourished by all 3 indicators (57.7%) while a large majority were stunted (92.6%). Only 3 subjects (1.8%) had no additional (i.e. to MUAC < 115 mm) anthropometric deficit.

Table 1 summarises the weight-for-height z-score (WHZ), weight for age z-score (WAZ), TSF for age (TSF/A) z-score and AFI for age (AFI/A) z-score at discharge. No study subjects (*n = 0*) were overweight or obese or had TSF/A > 2 z-scores or AFI/A > 2 z-scores at discharge.

**Table 1:** Anthropometry measures at discharge, (Lilongwe District, Malawi Feb 2011 to March 2012)

	Count (%) in each z-score class				Summary	
Index	z < − 2	- 2 ≤ z ≤ 2	z > 2	All	Median z	IQR
W/H	0 (0.0%)	163 (100.0%)	0 (0.0%)	163 (100.0%)	−0.50	− 0.98 to − 0.12
W/A	112 (68.3%)	51 (31.7%)	0 (0.0%)	163 (100.0%)	−2.37	− 2.91 to −1.87
TSF/A	8 (4.9%)	155 (95.1%)	0 (0.0%)	163 (100.0%)	−0.76	−1.35 to − 0.36
AFI/A	1 (0.6%)	162 (99.4%)	0 (0.0%)	163 (100.0%)	−0.65	−1.06 to − 0.29

**Fig. 2:**
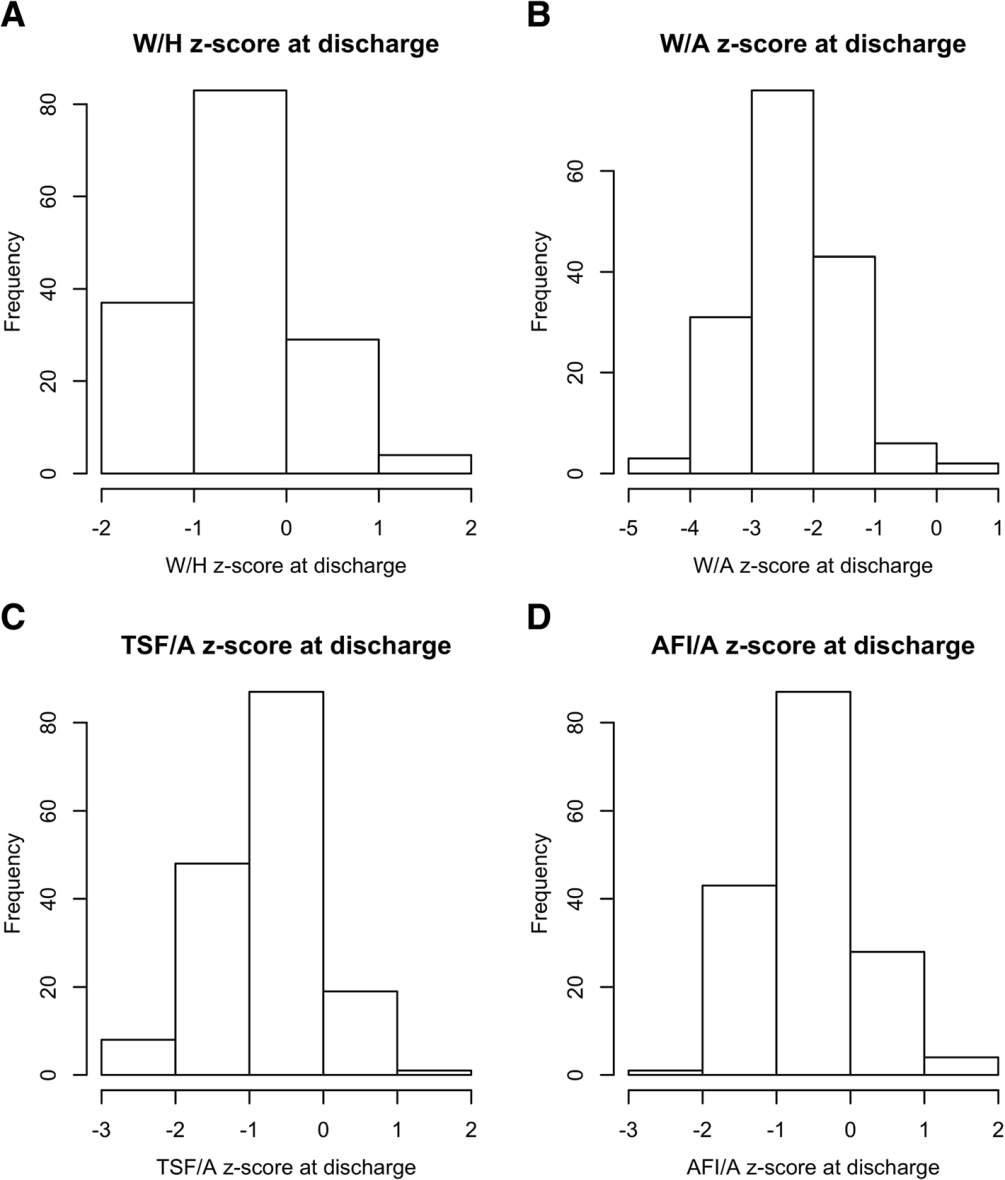
**a** Distribution of weight-for-height (W/H), **b** weight-for-age (W/A), **c** triceps skinfold-thickness-for-age (TSF/A), and **d** arm-fat-index-for-age z-scores at discharge (AFI/A). (Lilongwe District, Malawi Feb 2011 to March 2012)

Figure 2A shows the distribution of WHZ at discharge. No study subject was overweight or obese for height (*n = 0*) at discharge.

Figure 2B shows the distribution of WAZ at discharge. No study subject was overweight or obese for age (*n = 0*) at discharge.

Figure 2C shows the distribution of TSF/A z-score at discharge. No study subject had excess adiposity for age (*n = 0*) at discharge.

Figure 2D shows the distribution of AFI for age (AFI/A) z-score at discharge. No study subject had excess adiposity for age (*n = 0*) at discharge.

Table 2 summarises the TSF/A z-score at discharge for 3 classes of degree of stuntedness on admission, severe (HAZ < − 3), moderate (HAZ between <− 2 and − 3) and not stunted (HAZ equal or > − 2). No subject *(n = 0)* had a TSF/A > 2 z-scores at discharge.

**Table 2:** TSF/A z-score at discharge for three classes of stuntedness at admission (*n* = 163), (Lilongwe District, Malawi Feb 2011 to March 2012)

HAZ at admission	Median TSF/A at discharge	IQR	Maximum TSF/A at discharge
HAZ < − 3	− 0.70	− 1.09 to − 0.30	+ 1.08
−3 ≤ HAZ < − 2	− 1.04	− 1.58 to − 0.44	+ 0.27
HAZ ≥ − 2	− 1.16	−1.60 to − 0.62	+ 0.85

Table 3 summarises AFI/A at discharge for 3 classes of degree of stuntedness on admission as above. No subject *(n = 0)* had an AFI/A z-score greater than 2 at discharge.

**Table 3:** AFI/A at discharge for 3 classes of stuntedness at admission (n = 163), (Lilongwe District, Malawi Feb 2011 to March 2012)

HAZ at admission	Median AFI/A at discharge	IQR	Maximum AFI/A at discharge
HAZ < − 3	− 0.57	−0.82 to − 0.03	+ 1.20
− 3 ≤ HAZ < − 2	− 1.00	− 1.39 to − 0.60	+ 0.13
HAZ ≥ − 2	− 1.02	−0.34 to − 0.86	+ 0.56

**Fig. 3:**
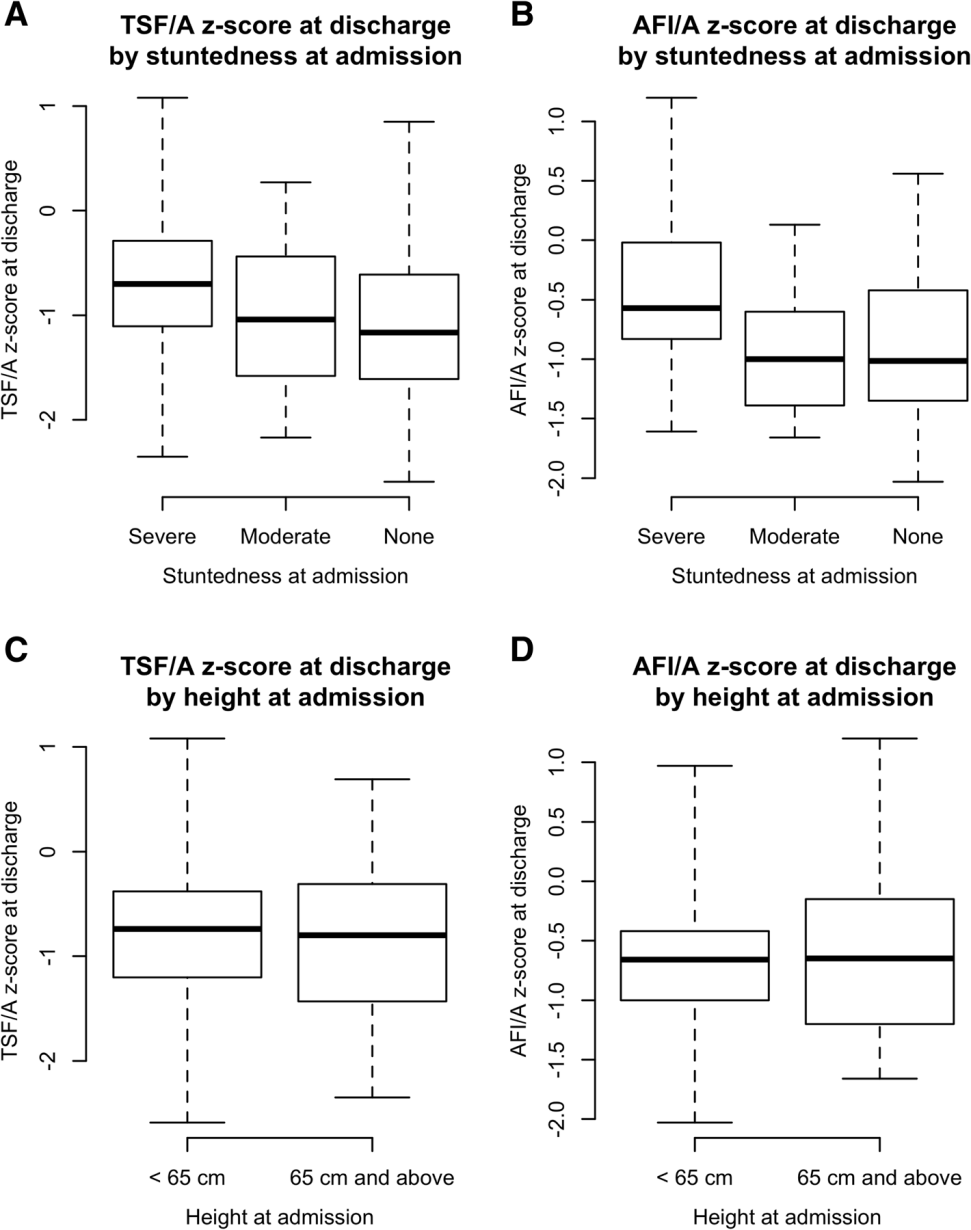
**a** & **b** Triceps skinfold-for-age (TSF/A) & Arm fat index-for-age (AFI/A) z-score at discharge by degree of stuntedness at admission. (Lilongwe District, Malawi Feb 2011 to March 2012). **c** & **d** Triceps skinfold-for-age (TSF/A & Arm fat index-for-age (AFI/A) z-score at discharge for two height classes at admission (Lilongwe District, Malawi Feb 2011 to March 2012). **a** to **d**: For the box plots presented in Fig. 3a to 3D, the box extends between the upper and lower quartiles with the thick line in the box marking the position of the median. The whiskers extend to 1.5 times the interquartile distance above and below the upper and lower quartiles

Figure 3a presents a box plot of TSF/A z-score for 3 classes of stuntedness at admission; none (HAZ > − 2), moderate stunting (HAZ < − 3 to − 2) and severe stunting (HAZ < − 3). There is a tendency towards a higher TSF/A z-scores for severely stunted children compared to non-stunted children (Kruskal-Wallis chi-squared = 9.0675, *p*-value = 0.0107), however no children (*n* = 0) had excess adiposity at discharge.

Figure 3B presents a box plot of AFI/A z-score for 3 classes of stuntedness at admission as defined above. Children admitted as severely stunted were discharged with a higher AFI/A ratio than moderately stunted or non-stunted children (Kruskal-Wallis chi-squared = 15.9810, *p* = 0.0003), however no (*n* = 0) children had excess adiposity at discharge.

Figures 3C presents a box plot for TSF/A for two classes of height at admission, less than 65 cm and equal or greater than 65 cm. There is no significant difference in TSF/A on discharge between the two height classes (Kruskal-Wallis chi-squared = 0.9219, *p* = 0.3370).

Figure 3D presents a box plot for AFI/A for two classes of height at admission as above. There is no significant difference in AFI/A on discharge between the two height classes (Kruskal-Wallis chi-squared = 0.0740, *p* = 0.7855).

## Discussion

Children aged between 6 and 59 months were enrolled without height exclusions into an outpatient programme for the treatment of SAM with MUAC less than 115 mm and discharged cured with a MUAC equal to or greater than125mm following treatment using RUTF. Weight for height, weight for age, triceps skinfold for age and arm fat index for age z-scores less than + 2 z indicated an absence of overweight, obesity or excessive deposition of fat in all children following treatment with RUTF. While there is a tendency for severely stunted children to be discharged with higher TSF/A z-scores and higher AFI/A z-scores, none became overweight or exhibited excess adiposity. There were no significant differences observed in these indices between children with heights less than 65 cm compared to taller children. These results should allay concerns that children either with a height less than 65 cm or with severe stunting will become overweight or obese as a result of treatment with RUTF in the outpatient setting using WHO recommended MUAC admission and discharge criteria [5].

It is known that using MUAC less than 115 mm to identify SAM in this age group leads to the enrolment of stunted children [7–9]. This is supported by the present study; over 90% of children with MUAC < 115 mm also having a HAZ < − 2 z-scores. Data for this study group also indicated that when a discharge criterion of 125 mm is used to define cure, children with a height of less than 65 cm are equally able to able to reach this criterion as taller children, albeit with longer lengths of treatment [20].

The development of non-communicable diseases in later life has been linked to the development of markers for metabolic syndrome in children [27]. Metabolic syndrome has not been defined for children younger than 6 years [18], however for all age groups where it has been defined, obesity is a requisite component. If all children, irrespective of height or stuntedness are able to reach the discharge criteria satisfactorily and not develop obesity then this study suggests that, for children with SAM and recruited by MUAC, treatment with RUTF does not contribute towards obesity as a risk factor for metabolic syndrome. Studies using other markers of metabolic syndrome such as leptin have demonstrated that after 4–10 weeks of treatment with RUTF, leptin levels are lower than those of normally nourished children and approximately 7 times lower than the threshold for predicting metabolic syndrome [29, 30].

It has been reported in other studies that body composition and physical outcomes suggest a potentially greater risk for non-communicable diseases in later life and that there are enduring effects of SAM on growth, body composition and physical function, however treatment for SAM resulted in catch up growth and an absence of cardiometabolic markers for NCD. The long term negative effects of SAM need to be considered alongside the effects of low birth weight and stunting in young children, urbanisation and access to diets high in fat and sugar in later life [13, 14] and other drivers of malnutrition such as poverty, food insecurity and lack of access to adequate healthcare [31].

Given that concurrent wasting and stunting in children carries a high risk of mortality and that MUAC additionally selects these children who are able to respond well to treatment with RUTF, it is appropriate that MUAC does select these children. Furthermore, the widespread use of MUAC in the community [32] and early recruitment of children into treatment programmes may result, not only in shorter episodes of treatment [20] and the potential for recovery without adverse cardiometabolic effects [13], but may translate into mitigating the enduring effects of SAM in later life. In addition to early treatment, improved post discharge care may also minimise and long term adverse outcomes for children surviving SAM [13, 33, 34].

It is known that different ethnicities express various phenotypes in relation to muscle and fat mass ratios [7, 8]. Further studies are needed to identify whether similar changes to those found in this study occur in other regions and body shape phenotypes following treatment for SAM with RUTF.

## Conclusions

Children aged from 6 to 59 months enrolled in outpatient programmes for the treatment of SAM with MUAC less than 115 mm and discharged cured with a MUAC equal or greater than 125 mm following treatment using RUTF did not become overweight or develop excess adiposity.

While there is a tendency for severely stunted children to be discharged with higher TSF/A z-scores and higher AFI/A z-scores, none of these children became overweight or exhibited excess adiposity. There were no significant differences observed in these indices between children with heights less than 65 cm compared to taller children. These results should allay concerns that children either with a height less than 65 cm or with severe stunting will become overweight or obese as a result of treatment with RUTF using recommended MUAC admission and discharge criteria.

While malnutrition at various life stages has been demonstrated to predispose infants and children to develop metabolic syndrome in later life, the treatment of children for episodes of SAM with RUTF does not appear to contribute to that risk. Children aged greater than 6 months with low MUAC should be targeted for treatment independent of their respective degree of stunting as defined by their height-for-age or height at admission.

## Abbreviations

AFI: Arm Fat Index
AFI/A: Arm Fat Index for Age
CMAM: Community based Management of Acute Malnutrition
DSM: Dried Skimmed Milk
FANTA: Food And Nutrition Technical Assistance
FHI 360: Family Health International
HAZ: Height for Age z score
MUAC: Mid Upper Arm Circumference
RUTF: Ready to Use Therapeutic Food
SAM: Severe Acute Malnutrition
TSF: Triceps Skinfold Thickness
TSF/A: Triceps Skinfold Thickness for Age
WAZ: Weight for Age z score
WGS: World Health Organisation Growth Standards
WHO: World Health Organisation
WHZ: Weight for Height z score
